# Radiographic Changes in Colombian Asbestos Factory Workers

**DOI:** 10.5334/aogh.2634

**Published:** 2020-01-03

**Authors:** Arthur L. Frank, Guillermo Villamizar, Jose Gabriel Bustillo Pereira

**Affiliations:** 1 Dornsife SPH, US; 2 La Fundacion Colombia Libre de Asbesto, CO; 3 Fundacion Universitaria Juan N Corpas Medicina, CO

## Abstract

**Background:**

Until recently, Colombia has been a country actively using asbestos. A major factory in Bogota manufactures friction products.

**Objective:**

To determine if the use of chrysotile asbestos in a friction products facility leads to workers developing disease.

**Methods:**

One hundred forty-eight factory workers, former workers, or retirees volunteered for X-ray and pulmonary function testing after informed consent. X-rays were read by two readers who needed to agree on positive findings.

**Results:**

Nineteen of the 148 X-rays had changes consistent with the known prior exposure to asbestos, mostly parenchymal in nature. Pulmonary function was not altered in most of the studied population.

**Conclusion:**

Asbestos disease is clearly present among Colombian asbestos factory workers, as is seen in other exposed populations around the world.

## Introduction

The use of asbestos in any form in past use in thousands of products has been known to cause a variety of non-malignant and malignant diseases [1]. More than 60 countries have now banned the use of asbestos [2], including Colombia in 2019. As noted by the work of Takahashi [3], asbestos-related disease has been found in many countries of the world.

Today most of the world’s asbestos comes from only a few countries, including Russia, Kazakhstan, and China [4], and is primarily used in construction materials, such as cement, although safer substitutes are available [5]. Cement plants around the world using asbestos have been shown to cause disease among workers in Brazil, Canada, and Egypt, among other places [6,7,8]. Asbestos use in manufacturing and construction in many settings has also been documented to cause disease [9].

In Colombia, which has had an active chrysotile mine that is now to be closed, asbestos has been used mainly for cement and vehicle brakes. Water tanks have been made from asbestos cement. There is little about asbestos disease in the scientific literature from Colombia. One flawed study of Colombian asbestos-exposed workers [10] reported no disease in an asbestos cement plant. The average latency was short, about twelve years, and only X-rays with an ILO reading of 1/1 or higher were considered possibly positive, although the ILO guidelines themselves clearly note that films read as 1/0 or higher are to be considered abnormal. They also stated 1/1 was only suggestive of disease. The level of exposure to workers was about 1.33 fibers/cc/year, a level that has been shown to be many times that which is needed to significantly increase the risk of mesothelioma [11].

Others in Colombia have evaluated exposures to asbestos in garage settings and have found elevated workplace levels in both auto and truck garages [12,13]. A recent study found evidence that suggests a mesothelioma cluster in Sibaté, a small town close to Bogotá where an asbestos factory has operated since 1942 [14].

## Methods and Materials

Free examinations were offered in a plant of approximately 500 active workers at a Colombian asbestos-utilizing facility making automotive friction products with chrysotile, as well as former workers and retirees. All who participated signed a consent form which had been developed by an NGO, FundClas, and approved by the Union representing the workers. Work histories were administered to all workers who consented to be examined. They were evaluated with PA and lateral chest X-rays and pulmonary function testing. Each participant was sent a personal letter with their radiographic findings and was told of their PFT findings. The group examined should therefore be considered self-selected.

All X-rays were read by two of the authors, and for an X-ray to be considered positive both readers had to agree. One reader is an experienced occupational physician and the other a pulmonologist.

## Results

One hundred forty-eight X-rays were evaluated from the workers of this one facility from the workforce of about 500, including administrative workers. All 148 who participated were production workers with exposure to asbestos. Of this number, 19 were found to have radiographic changes consistent with prior exposure to asbestos. Eleven were read as 1/0; five as 1/1; and one at 0/1 with bilateral pleural plaques; one at 1/0 with calcified plaques; and one with diaphragmatic calcified plaquing. Non-asbestos changes, such as emphysema, were noted in some of the one-hundred forty-eight films but not otherwise recorded. There was no evidence in these workers of any lung cancers or mesotheliomas.

The time of exposure for all workers and those that were positive can be found in Table 1. Most workers who developed disease did so within 20 years of first exposure.

**Table 1:** Prevalence of Asbestos-Related Pulmonary and Pleural Radiographic Abnormalities by Latency since First Exposure among Workers in a Friction Products Plant, Colombia, South America.

Latency (Yrs)	No. Workers Examined	No. Workers Positive	Prevalence (%)

4–5	2	0	0
6–9	13	0	0
10–14	19	5	26.3
15–19	27	7	25.9
20–24	58	6	17.2
25–29	24	1	4.1
30–34	5	0	0

Pulmonary function testing for most of those examined was normal, not a surprising finding because pulmonary function tests results do not correlate well with radiographic changes, especially at low profusion [15,16]. Changes were usually thought to be related to smoking.

The latency period for the workers exposed was between 4 and 34 years, 46 having exposure between 10 and 19 years, and 87 with more than 20 years. Only 17 had less than 10 years of exposure.

## Discussion

At this factory at least one mesothelioma has been noted in a worker in his forties, and at the National Cancer Institute in Bogotá there are multiple cases of mesothelioma treated each year at their clinical facility. Although perhaps not well documented, mesotheliomas, which are known only to be caused in such settings by asbestos exposure, are noted to occur throughout the country with its several asbestos cement plants and a chrysotile mine. These findings of asbestosis document, as has been shown in many other countries, that Colombian workers who are exposed to asbestos can and will develop disease from such exposures. In some studies, parenchymal disease is more prevalent than pleural disease [17,18], while in others, especially with low dose exposure, the opposite is noted. There are limitations to this study, but without doubt asbestos causes disease in Columbia, as it does elsewhere. These limitations include observing only one facility, self-selection of participants, and small numbers. Other workplaces measured in Columbia where X-rays have not been taken have levels rivaling or exceeding current US OSHA standards. Few other data are available from Colombia. These findings of asbestos disease deserve to be extended. Suitable substitutes exist for virtually every use of this material, including and especially for brakes and for cement.

## Conclusion

Asbestos causes measurable amounts of disease in Colombia among exposed workers, as has been found in many other countries.
